# ESPECTRA: Searching the Bipolar Spectrum in Eating Disorder patients

**DOI:** 10.1186/1471-244X-11-59

**Published:** 2011-04-14

**Authors:** Rodolfo N Campos, Jules Angst, Taki A Cordas, Ricardo A Moreno

**Affiliations:** 1 Researcher of the Mood Disorders Unit (GRUDA). Department and Institute of Psychiatry, Clinicas Hospital, University of Sao Paulo, School of Medicine, Sao Paulo, Brazil; 2 Zurich University Psychiatric Hospital, Zurich, Switzerland; 3 Eating Disorders Unit (AMBULIM) - Department and Institute of Psychiatry, Clinicas Hospital, University of Sao Paulo, School of Medicine, Sao Paulo, Brazil; 4 Director of the Mood Disorders Unit (GRUDA). Department and Institute of Psychiatry, Clinicas Hospital, University of Sao Paulo, School of Medicine, Sao Paulo, Brazil

## Abstract

**Background:**

Bipolar Disorder (BD) is a chronic, recurrent and highly prevalent illness. Despite the need for correct diagnosis to allow proper treatment, studies have shown that reaching a diagnosis can take up to ten years due to the lack of recognition of the broader presentations of BD. Frequent comorbidities with other psychiatric disorders are a major cause of misdiagnosis and warrant thorough evaluation.

**Methods/Design:**

ESPECTRA (*Occurrence of Bipolar Spectrum Disorders in Eating Disorder Patients*) is a single-site cross-sectional study involving a comparison group, designed to evaluate the prevalence of bipolar spectrum in an eating disorder sample. Women aged 18-45 years will be evaluated using the SCID-P and Zurich criteria for diagnosis and the HAM-D, YOUNG, SCI-MOODS, HCL-32, BIS-11, BSQ, WHOQoL and EAS instruments for rating symptoms and measuring clinical correlates.

**Discussion:**

The classificatory systems in psychiatry are based on categorical models that have been criticized for simplifying the diagnosis and leading to an increase in comorbidities. Some dimensional approaches have been proposed aimed at improving the validity and reliability of psychiatric disorder assessments, especially in conditions with high rates of comorbidity such as BD and Eating Disorder (ED). The Bipolar Spectrum (BS) remains under-recognized in clinical practice and its definition is not well established in current diagnostic guidelines. Broader evaluation of psychiatric disorders combining categorical and dimensional views could contribute to a more realistic understanding of comorbidities and help toward establishing a prognosis.

## Background

Bipolar disorder (BD) is a chronic and disabling condition associated with impairment of quality of life and occupational functioning. Diagnosing BD is a challenge due to its phenomenological complexity, variable course and correlates. The current classificatory systems (ICD-10 and DSM-IV) define diagnostic guidelines that have been subject to criticism for failing to represent clinical reality. As a result, reports based on these systems produce data that cannot be generalized for the population as a whole. The Bipolar Spectrum (BS) model suggests that presentations not officially recognized are part of a continuum and that different dimensions may be part of a single entity, e.g. BD/Unipolar Depression; BD/Schizophrenia; BD/Personality Disorder (Borderline) [1]. Although descriptions of BD's classic presentations are consistent in the literature, the expansion of the borders of the condition remains controversial [2]. The use of broader definitions results in a direct increase in the prevalence of the disorder. A review of the ECA study database (Epidemiological Catchment Area) showed that 6.4% of the general population met criteria for BS [3], while the original study reported a BD prevalence of 0.8%. Other researchers have found a prevalence ranging from 2.4 to 8.3% for the expanded forms of BS [4,5].

Although recent studies have shown that lifetime prevalence rates are higher than previously thought, BD remains under-recognized in routine clinical practice. A survey noted that 69% of patients with bipolar disorder reported an initial misdiagnosis, with more than one third experiencing a delay of 10 years or greater before receiving a definitive diagnosis of bipolar disorder [6]. Under-recognition of bipolar disorder has dramatic clinical consequences such as increased suicide risk, higher rates of substance use, iatrogenic worsening of long term course of the illness (due to antidepressant use) and higher economic costs [7]. Matza et al showed that the misdiagnosis of BD results in higher overall annual treatment costs due to inpatient, outpatient and drug expenses [8].

Comorbidity is the norm rather than the exception in BD and this is a major cause of misdiagnosis. Conditions that manifest simultaneously with BD tend to complicate the course and evolution through early onset, more severe episodes, acceleration of the cycles and suicidal behavior [9]. McElroy et al found that 65% of all patients with BD presented at least one lifetime comorbidity, 47% at least two and 24% at least 3 [10].

Eating Disorders (ED) are severe conditions often associated with other psychiatric disorders, in which the prevalence of at least one mood disorder can be up to 96% [11]. The enormous variation in the prevalence of comorbidities in this population partly reflects the different diagnostic criteria and instruments applied, although the strong relationship of these disorders is clear. A positive association between mood disorders and obesity has also been reported, and a recent study showed a high prevalence of BS in severely obese patients seeking surgical treatment [12].

Patterns of psychiatric comorbidity represent a possible strategy for improving recognition of bipolar disorder in patients presenting with depressive symptoms who may benefit from additional screening for bipolar disorder [8].

## Methods/Design

### ESPECTRA Project

ESPECTRA (*OCCURRENCE OF BIPOLAR SPECTRUM DISORDERS IN EATING DISORDER PATIENTS*) is a cross-sectional study designed to evaluate the prevalence of bipolar spectrum in an eating disorder sample.

The aim of the study is to examine a sample (ED) with a high prevalence of comorbidities, combining categorical and dimensional diagnostic perspectives, and their clinical correlates such as impulsivity, body image and quality of life. Evaluating clinical aspects beyond the phenomenological diagnosis should improve the detection of hidden bipolarity, determine degree of severity, and its consequences in multiple dimensions.

#### Design of ESPECTRA

The ESPECTRA study has a cross-sectional design involving a comparison group, and is to be conducted at a single site.

#### Subjects

Group 1: Inpatients or outpatients recruited from the Eating Disorder Unit of the Institute of Psychiatry of the Clinicas Hospital of the University of São Paulo, School of Medicine, are to take part in the study. Patients aged 18 to 45 years, clinically diagnosed with Anorexia Nervosa, Bulimia Nervosa or Eating Disorder Not Otherwise Specified will be selected for interview. The diagnosis shall be re-evaluated according to the DSM-IV-TR, and Binge-Eating Disorder patients will be excluded. Absence of other comorbidities is an exclusion criterion.

Group 2: The control group shall consist of outpatients recruited from the general gynecology clinic of the Clinicas Hospital of the University of São Paulo, School of Medicine. Based on medical records, women aged 18 to 45 years will be invited for interview. Those without Bipolar Disorder and with an Eating Disorder according to the DSM-IV-TR are eligible to participate in the study. Women with modified diagnosis of Premenstrual Dysphoric Disorder [13] will be excluded.

The protocol was approved by the local Research Ethics Committee, and conformed to the 2008 Helsinki Declaration (Ethics Committee - CAPPesq - Comissão de Ética para Análise de Projetos de Pesquisa do HCFMUSP - protocol number: 1154/09).

Written Informed Consent shall be obtained from all participants prior to any study-related activities.

#### Clinical Assessment

Standard data on demographics, general medical history, previous psychiatric treatment and family history of psychiatric disorders shall be collected. Specific information on laxative and amphetamine use as well as use of health resources and chronology of comorbidities are to be evaluated. Patients will be interviewed using the Structured Clinical Interview for DSM-IV Axis I Disorders (SCID-I) [14].

A supplementary evaluation of the Bipolar Spectrum (BS) will be carried out according to the Zurich criteria [15], considering hypomanic episode duration of at least 1 day and no exclusion criteria, such as: hypomania induced by antidepressant, substances (stimulants, alcohol, etc) and other causes (corticosteroids, brain lesion).

a) Hypomanic Syndrome:

- Euphoria, irritability or overactivity;

- Have personally experienced problems or received comments from others that something must be wrong with them (consequences)

- Present at least three out of seven signs and symptoms of DSM-IV hypomania

b) Hypomanic Symptoms:

- Episode of at least two hypomanic symptoms without consequences

The Bipolar Spectrum is defined as:

1) BD I: Major Depressive Episodes associated with mania;

2) BD II: Major Depressive Episodes associated with hypomanic syndrome (a) or hypomanic symptoms (b);

3) BD *minor*: dysthymia, minor depression or recurrent brief depression associated with hypomanic syndrome (a) or hypomanic symptoms (b);

4) Pure Hypomania: hypomanic syndrome (a) without diagnosis of depression and hypomanic symptoms only.

• Depressive and manic symptomatological assessment is to be conducted using the Hamilton Depression Rating Scale (HAM-D) [16], the Young Mania Rating Scale (YMRS) [17], the SCIMOODS [18,19] and Hypomania Checklist (HCL-32) [20,21] for current and lifetime symptoms.

• Quality of life and social adjustment: measured with the WHOQoL [22] and Social Adjustment Scale [23]. These instruments have previously been translated and validated in Portuguese [24,25]. WHOQOL is a self-report 100-item scale comprising 6 domains: physical, psychological, independence level, social, environment and spirituality. The Social Adjustment Scale is a self report 54-question instrument that measures instrumental and expressive role performance over the past two weeks.

• Impulsivity: Barrat Impulsiveness Scale (BIS-11) [26] evaluates impulsiveness in three domains: motor, lack of planning and attention. It is a 30-item self-rated Likert scale and has a Portuguese version [27].

• Body Image: Body Shape Questionnaire [28] evaluates concerns with body shape and obsession and self- depreciation due to the physical appearance. There is also a Portuguese version of the questionnaire available [29].

The participants shall be evaluated with the diagnostic instruments and symptoms rating scales without any kind of intervention.

All assessors had reliability training for applying the instruments and the diagnostic assessment (SCID-I and Bipolar Spectrum evaluation) is to be applied to all participants by the same investigator (RNC)

#### Primary outcomes

The main outcome is to determine the prevalence of bipolar spectrum disorders in ED patients of a tertiary-care hospital.

#### Secondary outcomes

With the aim of determining clinical correlates useful in diagnostic assessment, the following will be evaluated:

-Comorbidity profile;

- Quality of life and social adjustment;

- Impulsivity;

- Body image;

- Use of health services.

#### Planned Analyses

The sample will consist of patient groups with and without comorbidity that have BS, and prevalence rates will be estimated as the primary objective of the project.

Student's t test shall be employed to compare patient and control groups according to quality of life, social adjustment, impulsivity, body image and use of health resources for those variables presenting normality, which will be analyzed using Kolgomorov-Smirnov's test. Non-normal variables shall be submitted to the Mann-Whitney test. Qualitative variables of the groups wll be compared by Pearson's Chi-square test or Fischer's exact test.

Demographic and clinical information will be expressed as mean and standard deviation for continuous variables and as estimated frequencies for categorical variables.

A significance level of 5% will be adopted for all tests.

#### Sample Size

To ensure a precise estimate of the prevalence of BS, a 95% level of confidence and a 5% level of precision will be adopted. In order to calculate the sample size, an estimate of prevalence will first be required. Due to the large variation in prevalence of BD in the ED population (0% to 63.6%), an intermediate value of 20% was elected. Sample size (n) was calculated according to the following formula [30]:

where:

*n *= sample size,

*Z *= Z statistic for level of confidence,

*P *= expected prevalence or proportion

*d *= precision

This yielded n = 62, with 95% level of confidence and 10% level of precision.

## Discussion

The current classificatory systems in psychiatry use a descriptive approach since the etiology of most disorders remains unknown [31]. Despite the large number of studies on genetics and imaging techniques, phenomenology is still the core method of diagnosis. Robins and Guze outlined that diagnostic validity could be improved through precise clinical description, greater delineation of the syndromes from other disorders, laboratory studies, follow-up studies of outcome, and family studies [32]. A growing body of evidence has identified validators such as family history, demographic correlates, course of illness and concurrent symptoms (beyond the diagnostic criteria) [33] and a considerable amount of research has been focused specifically on determining whether mental disorders are best classified dimensionally or categorically.

The categorical model exhibits excessive diagnostic co-occurrence and raises the question as to whether these disorders really constitute distinct clinical entities. Despites its ease of use, the model is unable to effectively describe the interacting biological vulnerabilities and dispositions, or environmental and psychosocial events in a single diagnostic category [34]. It remains unclear if the dimensional model can provide a more valid description and classification of mental disorders. Core dimensions must be identified, measured and validated. How, and to what extent, these elements should be incorporated into the diagnostic system must also be determined [35].

The definition of BS is controversial and grounded in a dimensional model whose boundaries are still unclear. Although the proposed definitions differ on numerous aspects, there is a general consensus that three different presentations must underpin the BS definition [1]:

1- Hypomanic reactions to antidepressants

2- "Subthreshold" hypomania (below current diagnostic cut-offs)

3- Non-manic markers, such as: recurrent depression, family history of BD, early onset.

These different dimensions must be carefully assessed in future studies so that the controversies can give way to levels of evidence.

The ED patient group represents a high risk population for other psychiatric disorders in particular mood disorders. The prevalence of Major Depressive Disorder (MDD) for instance, can reach 81% in Anorexia Nervosa (AN) and 63% in Bulimia Nervosa (BN) patients [36]. Bipolar symptoms are also common in the ED population and McElroy et al reported that previous studies had found a prevalence of BD in ED patients ranging from 2.3% to 63.6% [37]. This heterogeneity reflects the different definitions of BD and instruments used to assess the condition, in some instances representing the categorical full criteria diagnosis and in others, subsyndromic cases.

Patients with both BD and ED tend to have a higher number of lifetime depressive episodes and greater psychiatric comorbidity, excluding eating and mood disorders [38]. The high comorbidity of BD and ED has yet to be fully explained. One hypothesis holds that they are different disorders and each represents risk factors for the other disorder, or even that they are similar final states of different etiological conditions. Another possibility is that the comorbid disorder is related through shared pathophysiological mechanisms [37].

The evaluation of lifetime comorbidities according to current classificatory schemes shows a high prevalence of psychiatric disorders that are not always "true comorbidities", highlighting the need to refine the conceptualization of mental disorders. Assessing comorbidity according to the official operational, criteria, and comparing with the broader dimensional model, the effect of these two approaches can be accurately estimated in a given population.

In this study, the diagnosis is considered to go beyond the classic symptoms, combining the categorical and dimensional approaches and taking into account other clinical correlates, in order to enhance both the reliability and validity of diagnosis. Establishing severity criteria based on less specific general parameters such as quality of life and use of health resources will help identify how the clinical profile is related to outcome.

BS is under-recognized in clinical practice [15] and comorbidities can represent major confounding factors delaying BS identification. The early onset of ED and its overlap with mood symptoms are also key points to consider in understanding the relationship of Mood Disorders and ED.

More comprehensive assessment and measurement of psychopathology can enhance operational diagnoses and lead clinicians to broader examinations [39]. Broader evaluation of psychiatric disorders beyond pure phenomenology and standardized categorical criteria can contribute to a more realistic understanding of comorbidities and improve the possibility of establishing prognosis.

## Competing interests

**RNC and TAC**: Declare no competing interests.

**RAM **has acted as a consultant to, and conducted research sponsored by, companies involved in the area of bipolar and depressive disorders (Servier, Bristol Myers Squibb, Solvay Pharma, Abbott, Astra Zêneca, Pfizer, and Roche).

**JA **has served on the advisory board for Eli Lilly & Company, Lundbeck, on the speakers' bureau for Eli Lilly & Company and AstraZeneca, and as a consultant for Sanofi Aventis.

## Authors' contributions

RAM made a substantial contribution to the conception and design of the study and was involved in revising the manuscript for key intellectual content.

RNC contributed to the study conception and design and provided clinical assistance.

TAC and JA contributed to the discussion on the methodology and the key points involved in the background of the study.

## Pre-publication history

The pre-publication history for this paper can be accessed here:

http://www.biomedcentral.com/1471-244X/11/59/prepub
